# A machine learning approach to predict pancreatic islet grafts rejection versus tolerance

**DOI:** 10.1371/journal.pone.0241925

**Published:** 2020-11-05

**Authors:** Gerardo A. Ceballos, Luis F. Hernandez, Daniel Paredes, Luis R. Betancourt, Midhat H. Abdulreda

**Affiliations:** 1 Knoebel Institute for Healthy Aging, University of Denver, Denver, CO, United States of America; 2 Diabetes Research Institute, University of Miami Miller School of Medicine, Miami, FL, United States of America; 3 Department of Surgery, University of Miami Miller School of Medicine, Miami, FL, United States of America; 4 Department of Microbiology and Immunology, University of Miami Miller School of Medicine, Miami, FL, United States of America; 5 Department of Ophthalmology, University of Miami Miller School of Medicine, Miami, FL, United States of America; Korea National University of Transportation, REPUBLIC OF KOREA

## Abstract

The application of artificial intelligence (AI) and machine learning (ML) in biomedical research promises to unlock new information from the vast amounts of data being generated through the delivery of healthcare and the expanding high-throughput research applications. Such information can aid medical diagnoses and reveal various unique patterns of biochemical and immune features that can serve as early disease biomarkers. In this report, we demonstrate the feasibility of using an AI/ML approach in a relatively small dataset to discriminate among three categories of samples obtained from mice that either rejected or tolerated their pancreatic islet allografts following transplant in the anterior chamber of the eye, and from naïve controls. We created a locked software based on a support vector machine (SVM) technique for pattern recognition in electropherograms (EPGs) generated by micellar electrokinetic chromatography and laser induced fluorescence detection (MEKC-LIFD). Predictions were made based only on the aligned EPGs obtained in microliter-size aqueous humor samples representative of the immediate local microenvironment of the islet allografts. The analysis identified discriminative peaks in the EPGs of the three sample categories. Our classifier software was tested with targeted and untargeted peaks. Working with the patterns of untargeted peaks (i.e., based on the whole pattern of EPGs), it was able to achieve a 21 out of 22 positive classification score with a corresponding 95.45% prediction accuracy among the three sample categories, and 100% accuracy between the rejecting and tolerant recipients. These findings demonstrate the feasibility of AI/ML approaches to classify small numbers of samples and they warrant further studies to identify the analytes/biochemicals corresponding to discriminative features as potential biomarkers of islet allograft immune rejection and tolerance.

## Introduction

The prospects of artificial intelligence (AI) and machine learning (ML) applications in biomedical research are exciting because they promise to unlock new information from the ever-expanding datasets being generated through high-throughput approaches in various areas of research. However, the current parsimonious usage of AI/ML in the biomedical field remains limited to medical diagnosis and disease staging-type applications [1, 2]. The Food and Drug Administration (FDA) recently recognized in a public statement on Software as a Medical Device (SaMD) released on January 28^th^, 2020 that “AI/ML technologies have the potential to transform healthcare by deriving new and important insights from the vast amounts of data generated during the delivery of healthcare every day”. However, by placing the emphasis on making sense of big data, this view by the FDA falls short of the initial purpose of AI/ML, which has been defined as to emulate the approach to problem-solving by the human brain. In fact, learning from incremental small amounts of data is closer to the way humans learn. Lake and colleagues used the Bayesian Program Learning method to demonstrate this concept by training a machine to reach a human performance-like level in a one-shot approach to learning a specific classification task [3]. Thus, AI/ML can and should be applied in various areas of biomedical research involving big as well as small data. The analysis presented in the current report demonstrates this notion in the context of a relatively small dataset generated using a focused metabolomics approach in pancreatic islet transplant mouse recipients.

Our research program is focused on the immunobiology of pancreatic islets during the development of diabetes and after islet transplant to treat patients with autoimmune type 1 diabetes. It is generally recognized that early detection is key in preventing diseases and achieving superior treatment efficacy, particularly, in chronic slow-progressing conditions such as diabetes. Early detection of pathological processes that lead to diabetes establishment would allow timely therapeutic interventions to halt/slow down diabetes progression. Similarly, identifying early changes in the functional and immune status of transplanted islets would prompt prevention of the islet graft deterioration or immune rejection before too late. Notably, early detection can be facilitated by biomarkers that report on the disease progress and the potential immune reactivity against tissue/organ transplants including pancreatic islets. Indeed, the type 2 diabetes biomarker glycated hemoglobin A1C (HbA1c) is routinely used to diagnose patients and to make decisions on when to initiate anti-diabetic treatments in patients. It is also used to assess the treatment efficacy. However, there is currently no such biomarker(s) for type 1 diabetes that can report early on the disease pathogenic stage before clinical diagnosis; and while the presence of autoantibodies after type 1 diabetes diagnosis is confirmed retrospectively in >90% of cases, their presence in subjects considered at-risk for type 1 diabetes has limited prospective or predictive value in determining who will or will not progress to symptomatic type 1 diabetes and clinical diagnosis since only ~10% of subjects with a single autoantibody progress to become symptomatic; and some with multiple autoantibodies never progress to clinical diagnosis [4]. Consequently, due to the lack of reliable early biomarkers that can distinguish type 1 diabetes progressors from non-progressors, investigational therapies can only be initiated at the time of clinical diagnosis based on the appearance of symptoms. Unfortunately, initiating therapeutic intervention(s) at diagnosis where significant damage to a critical insulin-producing cell mass has already taken place undercuts the efficacy and promise of therapies with the potential to halt or slow down the disease progression if administered early enough. Notably, type 1 diabetes remains today the only autoimmune condition without an approved immune modulatory therapy [5–8]. Similarly, there are currently no reliable biomarkers that can report in a timely fashion on the functional and immune status of pancreatic islet transplants in the treatment of type 1 diabetes; this prevents timely therapeutic intervention when needed during rejection episodes. This is unfortunate considering that the clinical experience has demonstrated islet transplant to be an effective form of cell replacement therapy of type 1 diabetes [9–11]. Although, the efficacy of islet grafts can also be limited on the long-term by various factors that lead to graft failure, with immune-mediated rejection being a major contributor [12]. Therefore, the lack of reliable early biomarkers in the context of both type 1 diabetes development and islet transplant therapy puts potentially efficacious preventive and therapeutic measures at a significant disadvantage.

Motivated by this critical need for early biomarkers of type 1 diabetes and graft rejection in islet transplantation, we have recently demonstrated the feasibility of localized metabolomics and proteomics analyses in the immediate microenvironment of islets to facilitate the identification of locally enriched early biomarkers in both contexts [13, 14]. These studies were made possible through the recent advances in methods to increase analyte separation by gas and liquid chromatography and to enhance their detection by mass spectrometry (e.g., GC-MS and LC-MS/MS) in combination with the experimental approach of islet transplant in the anterior chamber of the mouse eye (ACE), which uniquely allows direct access to the local microenvironment of ACE-transplanted islets as represented in the aqueous humor [15–17]. In the present report, we used the same ACE-platform in combination with another powerful and sensitive analyte separation and detection approach, micellar electrokinetic chromatography and laser induced fluorescence detection (MEKC-LIFD) [18], to analyze aqueous humor samples in the context of immune-mediated rejection and tolerance of islet allografts [19]. We created a locked software based on the support vector machine (SVM) method for pattern recognition in electropherograms (EPG) generated by MEKC-LIFD to predict whether a transplanted animal had rejected or tolerated its islet allografts, or whether it had not been transplanted altogether. These predictions were made based only on the peak heights in the aligned whole EPGs obtained in blinded aqueous humor samples from a total of 22 mice that were transplanted and either rejected or tolerated their islet allografts, or not transplanted as naïve controls. The results demonstrated a 21 out of 22 positive classification score (95.45% accuracy) among the three sample categories and 100% prediction accuracy between the rejecting and tolerant recipients.

## Materials and methods

### Animals

All animal studies in this report were specifically approved by the University of Miami’s Institutional Animal Care and Use Committee (IACUC; protocol # 17–171). Mice on the C57BL/6J (B6) and DBA/2 backgrounds were obtained from Jackson Lab (Bar Harbor, ME, USA) and were housed with free access to food and water under the supervision of the University of Miami’s Department of Veterinary Resources (DVR). Animal welfare was evaluated throughout the studies by DVR personnel and the study team members. Measures for humane euthanasia are in place in all IACUC approved protocols including for this study. The animals did not exhibit any signs of pain or distress in association with the performed procedures in this report. After reaching the experimental endpoint, the animals were euthanized, and aqueous humor and tissue samples collected for later analysis. Euthanasia was either by carbon dioxide or anesthesia (by isoflurane) overdose followed by cervical dislocation, as recommended by the American Veterinary Medical Association (AVMA) for the species.

### Islet isolation and transplantation in the anterior chamber of the eye (ACE)

**Fig 1:**
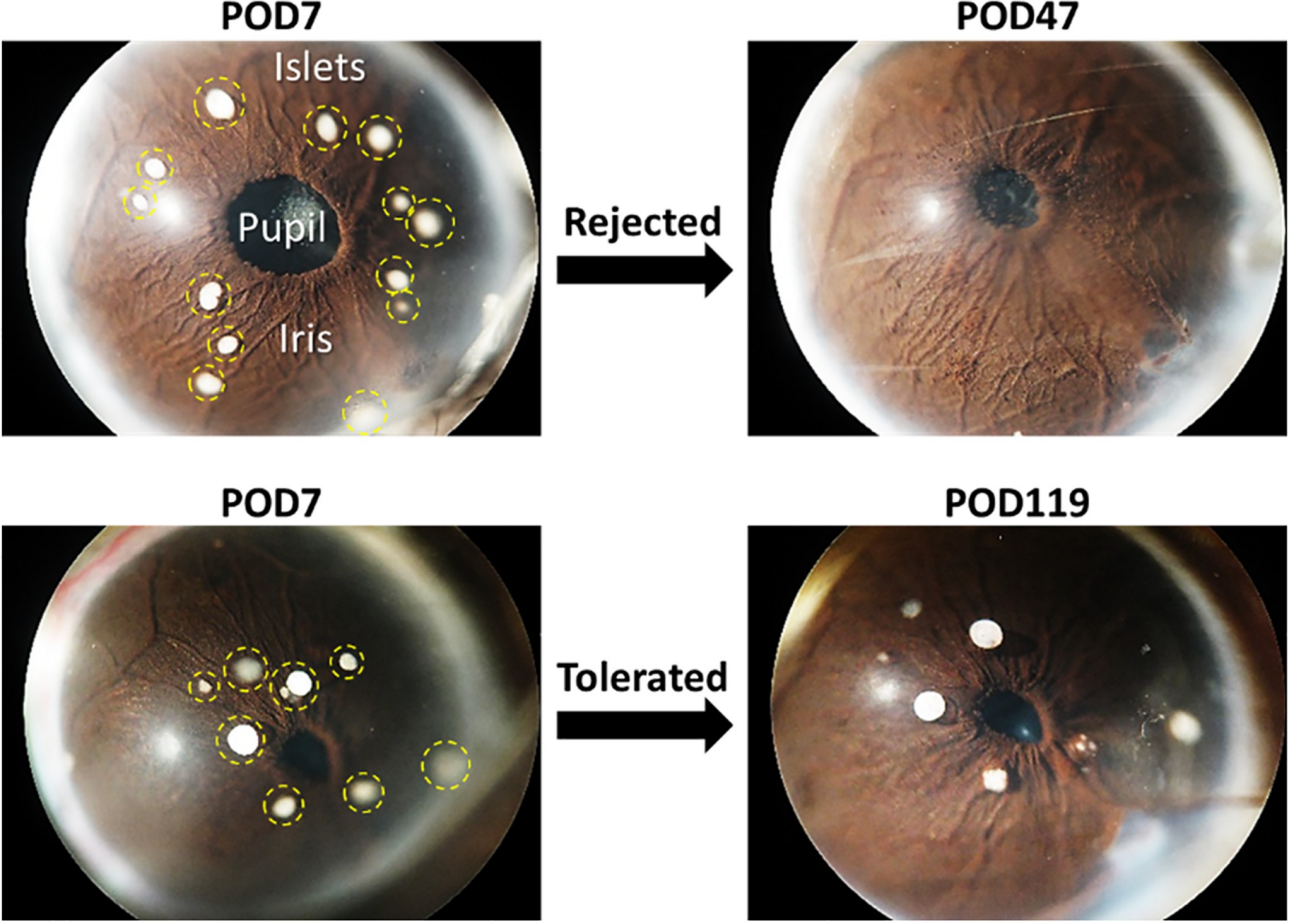
Pancreatic islet transplant in the anterior chamber of the eye (ACE) allows direct monitoring of the islet grafts and the collection of aqueous humor samples representative of the graft’s immediate microenvironment. Representative images of eyes with ACE-transplanted allogeneic islets from two recipient mice that either rejected (top) or tolerated (bottom) their islet allografts. Images on the left show the intact islet grafts (circled with yellow dashed lines) on day 7 after transplant (POD7). Images on the right show the eyes on either POD47 after complete graft rejection in the rejected mouse or POD119 in the tolerant mouse exhibiting intact islet allografts 4 months after the transplant.

Allogeneic pancreatic islets were obtained by enzymatic digestion of pancreata from DBA/2 donor mice followed by purification on density gradients using protocols standardized at the Diabetes Research Institute (DRI) Pre-Clinical Cell Processing and Translational Models Core, as previously described in detail [20]. The donor mice were euthanized by exsanguination while under general anesthesia (by ketamine/xylazine mix), as recommended for the species by the AVMA. After overnight culture, the isolated islets were implanted in the ACE (10–20 islet equivalents; IEQ) of one eye of fully-anesthetized recipient mice (by isoflurane), as previously described in detail [16]. In brief, the isolated islets were loaded into a reservoir connected to an ophthalmic cannula via tubing on one end and on the other to a motorized injection system operated by foot pedals. A small incision was made in the cornea at mid-point between its apex and the limbus and the islets were delivered into the anterior chamber of the eye on top of the iris where they engraft (see Fig 1) [16].

### Monitoring survival of the ACE-transplanted islets

Survival of the allogeneic ACE-transplanted DBA/2 islets was assessed by quantitative volumetric measurements of the individual islets based on their backscatter (reflection) of a 633 nm laser, as previously described in detail [15, 17, 21]. In brief, islets engrafted on top of the iris were mapped in digital images of the eye acquired during the first week after transplantation and the same islets were revisited during the longitudinal imaging sessions post-transplant. The islet survival was documented by direct visualization and assessment of their structural integrity in high-resolution digital images and by confocal micrographs acquired in the backscatter/reflection mode. Three-dimensional (3D) confocal micrographs of individual ACE-transplanted islets in each mouse were acquired using 20x magnification, 0.5 numerical aperture (NA) water immersion objective in z-stacks spanning the full height of each islet. The quantitative analysis of the individual islet volumes was performed in 3D using *Volocity* software (PerkinElmer; OH, USA), as previously described [17]. When the islet graft in a mouse lasted more than 100 days post-transplant (>Post Operation Day (POD)100) with the average volume of its individual islet grafts remaining ≥70% relative to their respective baselines, the graft in that mouse was considered tolerated [19]. A mouse was considered rejected when the average volume of its islets decreased <70% before POD100 [17]. Typically, mice rejected their intraocular islet grafts within 3–4 weeks post-transplant [17, 19]. Aqueous humor samples were collected at the time a recipient mouse was deemed to have rejected or tolerated its islet allograft as defined above (see Fig 1).

### Collection of aqueous humor samples

Samples of aqueous humor were collected from all mice following the procedure we previously described [13, 14]. Briefly, under isoflurane anesthesia the anterior chamber of the eye was penetrated laterally through the cornea with the tip (~40 μm diameter) of a borosilicate glass capillary tube (with 1.17 and 1.5 mm inside and outside diameter, respectively). Between 4 and 6 μL of aqueous humor were extracted by capillarity action and were frozen at -80°C for later analysis by MEKC-LIFD. Samples from rejecting recipients were collected at the time of rejection onset (±3 days). Samples from tolerant recipients were collected after confirming tolerance (i.e., >POD100).

### Sample derivatization

The aqueous humor samples were derivatized as reported elsewhere in detail [18]. In brief, a 2.5 mM Fluorescein Isothiocyanate Isomer I (FITC) in acetone solution was mixed 1:1 (V/V) with 20 mM Carbonate Buffer at pH 10.0. Two microliters of aqueous humor were mixed with 2 microliters of derivatizing solution. After 24 hours in the dark the samples were ready for MEKC-LIFD analysis.

### MEKC-LIFD analysis

MEKC-LIFD analysis was performed using a homemade instrument described elsewhere [18, 22]. In brief, a 60 cm long fused silica capillary with 25 and 350 μm inside and outside diameter, respectively, was filled with a Sodium Tetraborate (40 mM) and Sodium Dodecylsulphate (20 mM) buffer at pH 8.7. The samples were hydrodynamically loaded in the anodic end of the capillary by applying a –12 PSI pressure (vacuum) at the cathodic end of the capillary. An electric field was created by applying +27 KV at the anodic end of the capillary while keeping the cathodic end grounded. The bands were detected by a collinear detector equipped with a 488 nm continuous wave laser focused through a 64X, 0.85 NA objective on a 1 cm window made at 40 cm of the anodic end of the capillary by burning off the polyimide cover. The fluorescence was collected with the same objective through a long-pass filter centered at 505 nm and focused with a 10X eye piece on the window of a photomultiplier tube (PMT) to measure fluorescence intensity that will be converted into voltage in the electropherograms (EPG). The signal was collected at 40 points per second using a homemade software and data output in the form of voltage versus time was presented as EPGs. Unidentified peaks were referred to in this report as ‘untargeted peaks’. Ten (10) analytes/metabolites were identified by spiking, as previously described in detail [18]. Briefly, spiking consisted of derivatizing a 1 mg/mL solution of a known analyte by adding 5 μL of the derivatizing solution to obtain a standard solution. Since FITC was the limiting reactant, this solution yielded a single peak with negligible ghost peaks. Then a 1:1 v/v mixture of the sample and standard was prepared and run by MEKC-LIFD. All the peaks in the spiked samples decreased to about half of their amplitude in the corresponding un-spiked sample, but the peak amplitude of the standard (spiked) analyte increased allowing its identification. The identified peaks were referred to in this report as ‘targeted peaks’. The performance of our classifier was contrasted to other classifiers using targeted and untargeted peaks.

### Processing of electropherograms of untargeted peaks, peak detection and measurements, and statistical methods

**Fig 2:**
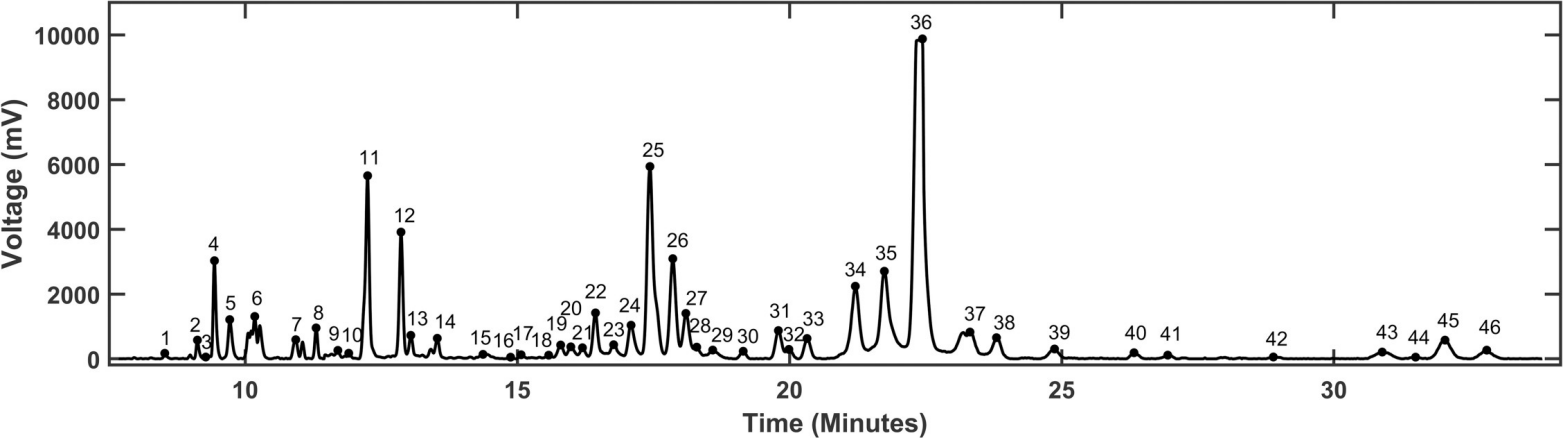
MEKC-LIFD generated electropherograms in aqueous humor show discrete peaks with various heights and widths. A representative electropherogram (EPG) pattern corresponding to one aqueous humor sample showing 46 detected peaks that could be measured and analyzed across all the aligned EPGs of other samples under the various conditions. Each peak corresponds to a putative analyte/biochemical whose identity can be determined as previously described [22, 26, 27]. The peak height and width can be used to measure the abundance levels of analytes and to contrast and compare the aligned EPGs from each sample.

The EPGs corresponding to untargeted/unidentified peaks were processed and aligned using a homemade software in MATLAB® by following procedures we reported on in detail elsewhere [23]. Data reduction, denoising, and region of interest detection were achieved using the Discrete Wavelet Transform (DWT) technique. Data reduction by 16 times was achieved by reducing 4x the DWT resolution level. We also used soft thresholding of detail coefficients at 5^th^ resolution level to reduce the noise. The detail coefficients at 7^th^ level were used for detecting activity zones with peak presence in order to discard the inactive zones in the EPGs; inactive zones represented ~25% of the data points in the acquired EPGs (note in Fig 2 that the peaks start to appear after minute 8). Moreover, a naive baseline correction method was applied detecting valleys and subtracting a polynomial approximation fitting these data points in the EPGs. Then, electropherogram alignment was performed using the methodology we previously described in ref. [23], where all the EPGs were coded to discrete characters using the first derivative coding approach. The positive derivative was coded as ‘m’, the negative derivative as ‘n’, and maximum peak points were coded as one of eight different characters depending on their height. The coded EPGs were aligned using Smith & Waterman optimized algorithm, which is frequently used in Bioinformatics for aligning amino acid or nucleotide sequences [24, 25]. This algorithm maximizes the reward function assigning positive scores to aligning of similar characters whereas mismatches and gap insertions are penalized with negative or lower values.

**Fig 3:**
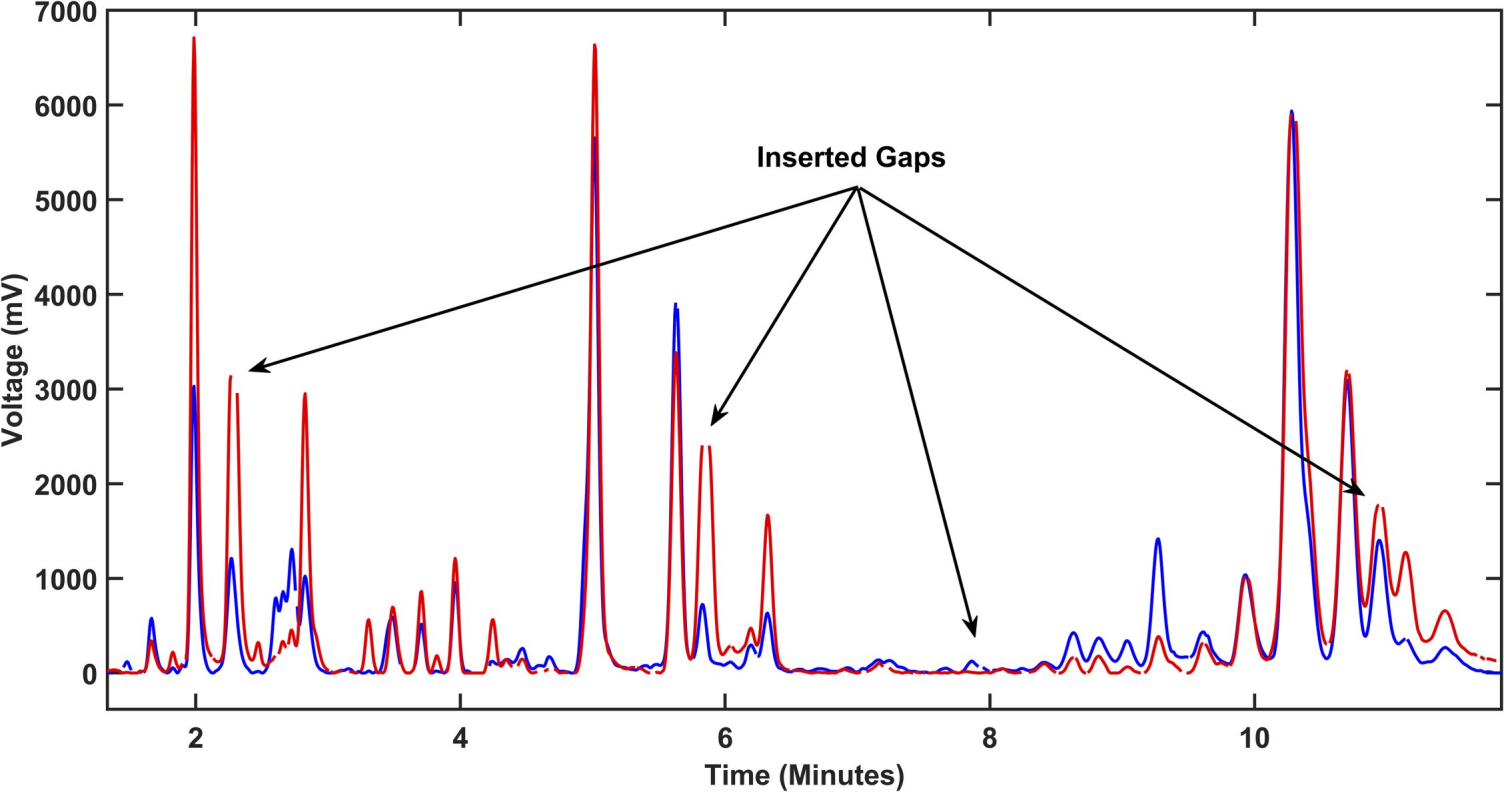
Software alignment of electropherograms for quantitative analyses. Alignment between two electropherograms (EPG) where one is used as a reference ‘key’ EPG (blue) to align the other (red). Gaps (examples highlighted by arrows) are inserted in both EPGs to obtain congruent migration time for all the peaks and achieve complete alignment (see Results/Discussion for more details).

**Fig 4:**
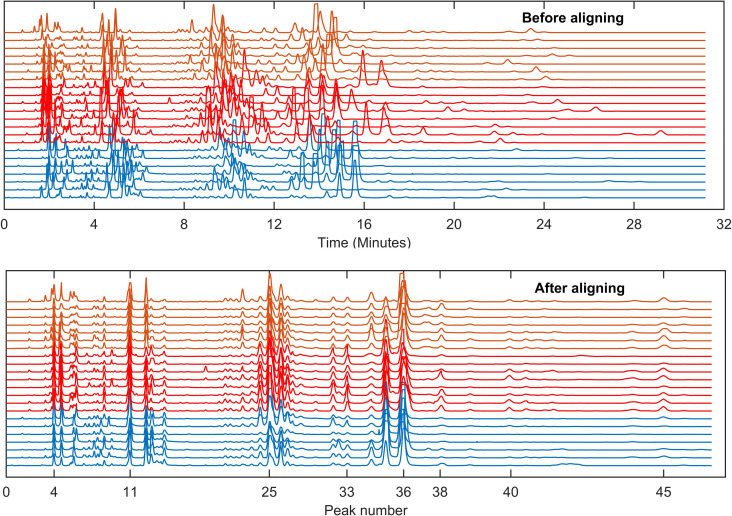
Electropherogram alignment is a fundamental step for automated peak measurement. Shown are the total 22 electropherograms (EPG) generated in the aqueous humor samples collected from transplanted mice that either rejected (orange) or tolerated (red) their islet allografts and from the non-transplanted controls (blue), before (top) and after (bottom) alignment. One was chosen randomly as the reference ‘key’ EPG to align the remaining 21 EPGs (see Results/Discussion). Migration times of each peak exhibited large dispersion before but congruency after the alignment. This process makes possible for the software to classify the samples across the three sample categories.

Next, we randomly chose an EPG from the dataset as a key coded reference EPG and used it to perform iterative pairwise alignment with the rest of the EPGs from the three sample categories (Fig 3). This method can result in some pair alignments with a few peaks not aligned with the corresponding ones. Our program, however, allows manual tagging of the corresponding peaks to force their alignment. After all peaks were coded in the EPGs and all the EPGs aligned to the key EPG (Fig 4), all peaks with heights above a predefined threshold (50mV) were detected in the reference ‘key’ EPG and all the corresponding values in the rest of EPGs were obtained from the remaining EPGs. All peak measurements were then arranged in a matrix. In our experiment, this matrix consisted of 22x46 elements (22 EPGs, 46 detected peaks) and was subjected to automated computational and statistical analyses and machine learning algorithms in order to classify the three categories of samples from which the EPGs were generated. A multi-comparison test for one-way analysis of variance (ANOVA) with Tukey-Kramer’s post-hoc test was performed.

### Processing of electropherograms of targeted peaks, peak detection and measurements, and statistical methods

We performed the above described electropherogram processing on targeted peaks (identified by spiking) but without alignment. The 10 (ten) targeted/identified peaks corresponded to the following metabolites: lysine, putrescine, arginine, glutamine, Gamma Aminobutyric Acid (GABA), glutamate, tryptophan, tyrosine, phenylalanine, and kynurenine. The peak of the standard (spiked analyte) corresponded to an 8.6 μM solution. Therefore, we calculated the μM concentration of each targeted/identified analyte in each sample by dividing its peak height (and/or the area) by the height (and/or the area) of the corresponding peak in standard/spiked 8.6 μM solution. For each analyte, the data were divided in three groups: non-transplanted controls, tolerant and rejected recipients. The three groups were compared by ANOVA followed by Tukey-Kramer Post-hoc multiple comparison test. For all the identified peaks, the ANOVA test showed statistically significant differences: lysine: F(2/22) = 11, p<0.001; putrescine: F(2/22) = 3.691, p<0.043; glutamine: F(2/22) = 7.784, p<0.003; arginine: F(2/22) = 12.32, p<0.0003; glutamate: F(2/22) = 41.8, p<0.0001; tyrosine: F(2/22) = 22.3, p<0.00001; phenylalanine: F(2/22) = 13.3, p<0.0002; GABA: F(2/22) = 21.6, p<0.0001; tryptophan: F(2/22) = 22.78, p<0.0001; kynurenine: F(2/22) = 7.6, p<0.003; and the kynurenine/tryptophan ratio: F(2/22) = 5.336, p<0.01. Finally, the classification software was run with these data.

### Sample classification

To discriminate among the three sample categories (rejected, tolerated and control) and classify whether a sample belonged to a rejecting or tolerant islet transplant recipient, we used a popular and effective machine learning approach highly efficient in small datasets, namely, support vector machine (SVM). This method can map the features to a higher dimension to find a hyperplane separating two classes by maximizing the distance between the hyperplane and special key samples from both classes near it (support vectors). SVM can create higher dimension representation depending on the chosen kernel, where each kernel defines the inner product in a different way. We used a linear kernel, then the hyperplane was found in the same space of the original features (without mapping to higher dimension). For comparative purposes, we also tested three more simple classifiers: Binary Tree, Naive Bayes, and KNN-4. For the SVM case, when the three sample categories were considered, ones-versus-one classification was applied, i.e. three binary classifiers were built, and the testing sample was assigned to the more frequent assigned class. Classification was tested by considering three versus two classes, and by contrasting them using two feature selection methods: all peak height measurements (i.e., untargeted peaks; whole EPG) versus targeted peaks selected by ANOVA. Peak selection consisted of applying ANOVA in a training sample set to choose as discriminative peaks only those which rejected the null hypothesis–that is, the mean heights for all the groups are equal–with a significance level of *p*<0.05. Leave-One-Out cross-validation (LOOCV) was also used for setting up the training and validation sets. Thus, the classifier was iteratively trained 22 separate times on all the data except for one sample and a prediction was made for that sample. The accuracy of the prediction/classification was reported as the average of the 22 results.

## Results and discussion

We investigated in this report whether it is possible to discriminate among transplant recipients that either rejected or tolerated their allogeneic pancreatic islet grafts using an ML/AI approach based solely on EPGs generated by MEKC-LIFD analysis in small samples of the immediate graft microenvironment. To gain access to these samples, we used our established ACE-platform [16] and collected microliter-size aqueous humor samples representative of the immediate microenvironment of the islets transplanted there. We previously demonstrated that the ACE-platform, not only allows direct access to the graft local microenvironment [13, 14, 19], but also enables the noninvasive monitoring of the graft’s fate and the quantitative assessment of its survival longitudinally (Fig 1) [15, 17, 19]. Thus, aqueous humor samples were collected under conditions of immune-mediated rejection or tolerance of the intraocular islet allografts, and MEKC-LIFD analyses were performed to assess their biochemical composition and for comparison of the corresponding EPGs (Fig 2) [18, 22, 26, 27].

**Fig 5:**
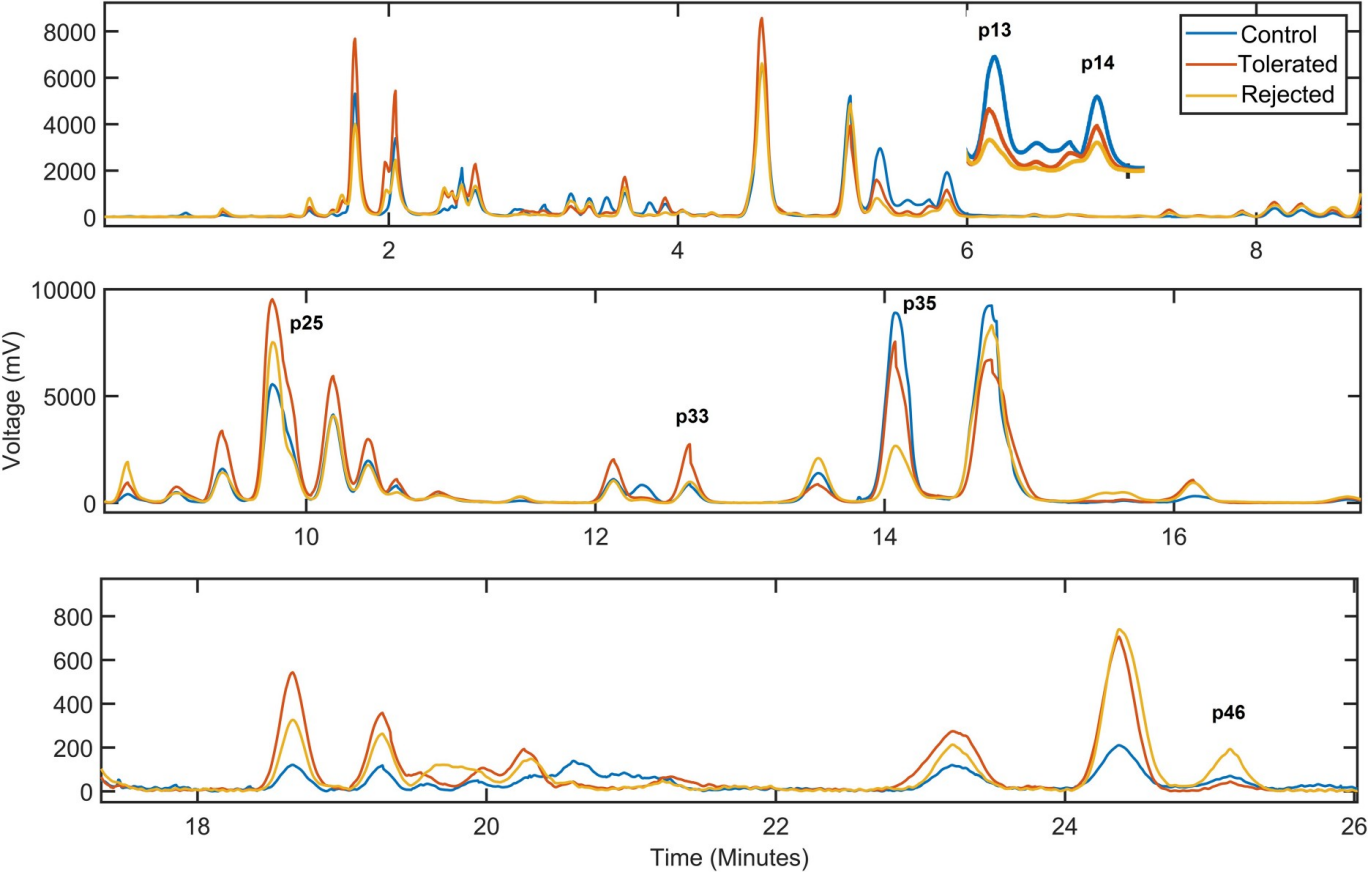
Electropherogram alignment enables the comparison of peaks and potential identification of biomarkers. Shown is a time-expanded view (along the X-axis) of the averaged electropherograms (EPG) derived in the three aqueous humor sample categories, i.e., from the non-transplanted controls and from transplanted mice that either rejected or tolerated their islet allografts. Comprehensive one-way analysis of variance (ANOVA) of height of the individual peaks (each corresponding to a putative analyte) identified 23 significant differences in peaks that clearly discriminated between the sample classes. Six of most significant peaks are labeled in the figure. Notably, these peaks correspond to putative analytes which can potentially be used as biomarkers of islet allograft immune-mediated rejection versus tolerance.

**Fig 6:**
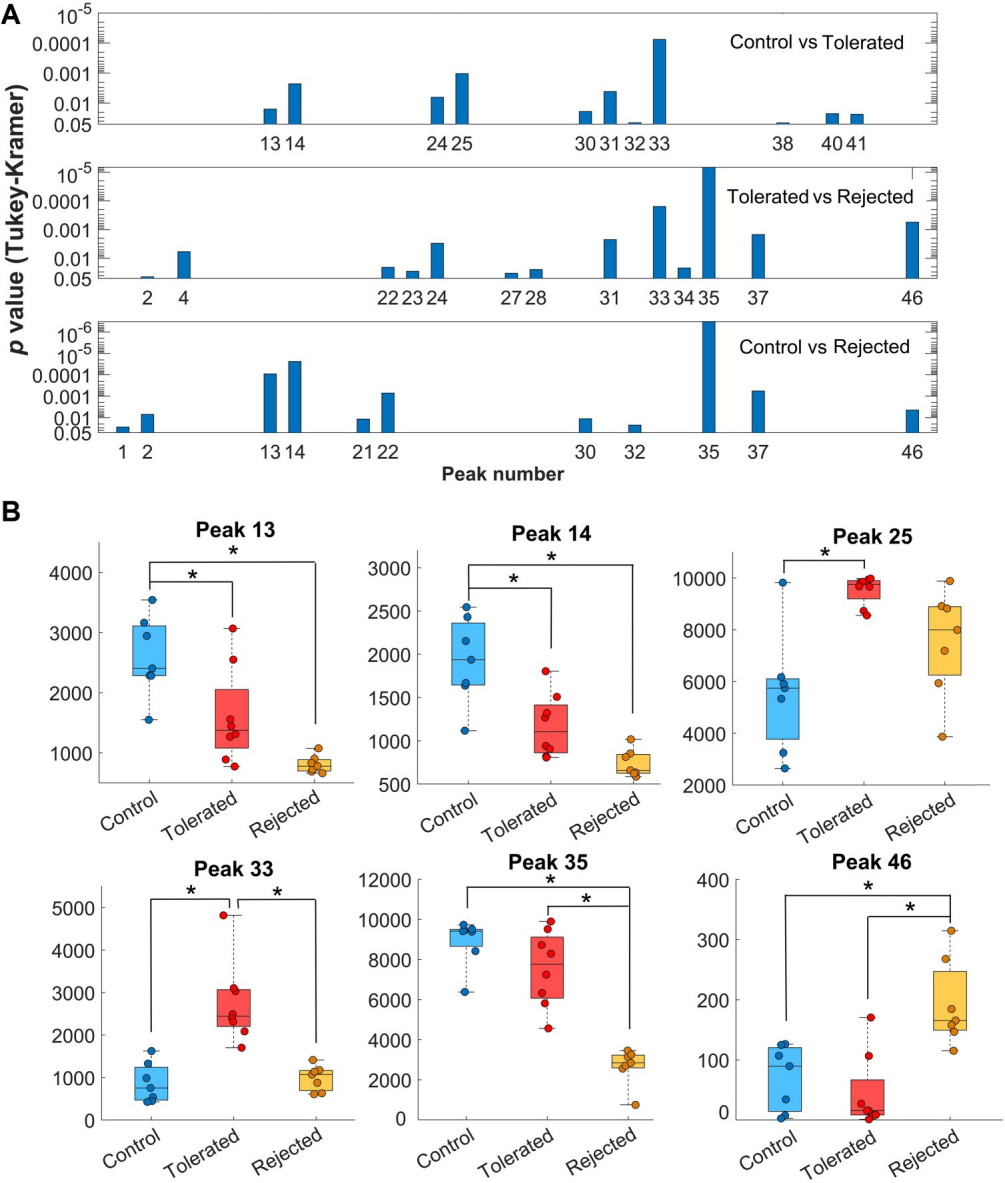
Several peaks in the electropherograms were identified with significant differences among the control, rejected and tolerated sample categories. **(A)** Distribution of *p* values from Tukey-Kramer post-hoc test comparing the indicated peaks in the electropherograms (EPG) obtained in the pairwise comparisons of control (*n* = 7), tolerated (*n* = 8), and rejected (*n* = 7) sample categories. The higher the bar the lower the probability of the null hypothesis that there was no difference between the heights of the compared peaks. **(B)** Height values of the peaks with the highest significance when comparing the EPGs obtained in the three sample categories. Data are shown as box and whiskers plots indicating the medians (horizontal lines inside each box) and the upper and lower quartiles with all data points shown as round symbols. Asterisks indicate significant differences (*p<0.00001).

Analysis of the EPGs allowed the identification of discrete peaks, each corresponding to a putative biochemical feature/metabolite, that could be compared across the EPGs of each individual mouse and to discriminate among the sample categories (i.e., rejected, tolerated, and non-transplanted controls). Alignment of the EPGs was necessary for measuring the heights of the peaks and their proper comparison (Figs 3 and 4). While, in the untargeted analysis, we did not determine the identity of the specific biochemicals (metabolites) corresponding to the unidentified/untargeted peaks, abundance measurements of such features based on their peak height and peak area values could be obtained from the EPGs [26, 27]. By averaging the peaks in the fully aligned EPGs of the samples in the same category and overlaying the corresponding averaged EPGs, evident differences in some of the peaks across the three sample categories were revealed (Fig 5). Notably, these discriminative peaks could potentially be of significant value as candidate metabolic biomarkers of immune-mediated rejection and tolerance of transplanted islets [14]. After comparative analysis of the peaks by ANOVA as described in Methods, we obtained 23 from 46 peaks that rejected the null hypothesis (degrees of freedom were respectively 19 and 2 within each of the sample categories and between the categories; *p*<0.05). These peaks were referred to here as ‘untargeted (ANOVA)’ in Tables 1 and 2. A Tukey-Kramer Post-hoc multiple comparison test revealed 11 peaks with different mean peak heights for the naïve non-transplanted “control” versus “tolerated” samples, 13 peaks for “tolerated” versus “rejected”, and 11 peaks for the “control” versus “rejected” sample categories. Distribution of *p* values resulting from each pairwise comparison of the discriminative peaks showed highly significant differences between the sample categories (Fig 6A). Peaks 13, 14, 25, 33, 35, and 46 had the highest significance in the pairwise comparisons among the three categories (Fig 6B). For example, peak 35 was the most discriminative between “tolerated” and “rejected” as well as the “control” versus “rejected” samples. Interestingly, while the height of peak 35 was reduced in the “rejected” group, that of peak 46 was increased in the same group; and peak 33 height was increased in the “tolerated” when compared to the “rejected” group. Thus, the putative analytes corresponding to these discrete peaks could potentially be identified, as we previously described [26, 27], and investigated further as candidate biomarkers of islet allograft rejection versus tolerance. Furthermore, similar studies conducted longitudinally in the same animals could also reveal the time evolution of such biomarkers [14], and may facilitate the prediction and classification of rejectors from non-rejectors in islet transplantation.

**Table 1:** Classification accuracies of the 3 sample categories (control, tolerated, and rejected) using the various LOOCV classifiers (including our SVM classifier) based on 46 untargeted peaks, 23 untargeted peaks selected by ANOVA, and 10 targeted peaks.

Classification accuracy (%)	Untargeted	Untargeted (ANOVA)	Targeted
**SVM**	**95.45**	90.90	78.26
**Binary Tree**	81.81	81.81	78.26
**Naive Bayes**	72.72	72.72	82.60
**KNN-4**	81.81	72.72	30.43

**Table 2:** Classification accuracies between 2 group categories (tolerated versus rejected) using the various LOOCV classifiers (including our SVM classifier) based on 46 untargeted peaks, 23 untargeted peaks selected by ANOVA, and 10 targeted peaks.

Classification accuracy (%)	Untargeted	Untargeted (ANOVA)	Targeted
SVM	100	100	100
Binary Tree	100	100	87.5
Naive Bayes	100	100	100
KNN-4	93.33	93.33	81.25

As stated above, we focused here on demonstrating proof-of-concept for the application of AI/ML to identify patterns/signatures of biochemical changes based on a small dataset of EPGs obtained in a limited number of biological samples, as would the case in a potential clinical application where prediction of whether a recipient of pancreatic islet transplant is at risk of rejecting their graft can be made based on a specific biochemical/metabolic signature in samples from that patient. A similar effort was recently reported by Kantzelmeyer and colleagues where they developed a classifier program using a SVM method to detect chronic antibody-mediated rejection (cABMR) in kidney transplant recipients based on data acquired by capillary electrophoresis and mass spectrometry of urine samples [28]. The authors trained their classifier on 24 samples from kidney transplant recipients with cABMR and 36 samples from recipients without cABMR, and they were able to detect recipients with cABMR with a misclassification score of only 2 out of 20 when combining their classifier with the proteomic marker CKD273. In the present study, we considered the peak height measurements as features in a Leave-One-Out cross-validation (LOOCV) scheme with a total of 22 EPGs using the SVM method with linear kernel as we described in Methods. We compared the performance of this SVM classifying model with other simple classifiers, namely, Binary tree, Naive Bayes, and KNN-4 under the three conditions (i.e., rejected, tolerated, and non-transplanted controls) using ***1)*** all peak measurements as classification features where the overall pattern of the 46 untargeted/unidentified peaks was recognized, ***2)*** a feature selection from untargeted/unidentified peaks by means of ANOVA, and ***3)*** the 10 targeted/identified peaks corresponding to metabolites with significantly different abundances in the three sample categories (see Methods). Table 1 shows estimation of the prediction accuracy of classifying a sample to the correct category (i.e., rejected, tolerated, or naïve control). Interestingly, the classification accuracy using our SVM model with all untargeted peaks (i.e., whole EPG) was better than both, those with untargeted peaks selected with ANOVA and the 10 targeted peaks; classification accuracy was 95.45% versus 90.90% and 78.26%, respectively (Table 1). This finding suggested that our classifier algorithm recognized patterns in the complete EPGs, inclusive of discriminative and non-discriminative peaks, which improved the accuracy of the classification. This highlights the potential for additional data mining and information extraction from the EPGs for biomarker discovery. Moreover, Table 2 shows accuracy estimates of classifying whether a sample out of the 22 belongs to either the "tolerated" or "rejected" group. These results suggest that the "tolerant" versus "rejected" classification is easier between two sample categories compared to three. Furthermore, our SVM and the Naive Bayes models classifiers detected with 100% accuracy samples from the "tolerant" and "rejected" categories with any of the configurations (i.e., untargeted peaks, untargeted peak selected by ANOVA, and targeted peaks).

These data indicated that the automated untargeted/unidentified peak selection tended to outperform the restricted peak selection either by ANOVA (untargeted) or targeted peaks in correctly classifying a sample among the three categories; and the Naive Bayes classifier performed similar to our SVM classifier when using two sample categories (rejected versus tolerant). This is an interesting observation with practical implications because targeted selection of peaks is more time- and effort-consuming than automated selection based on patterns. On the one hand, it is important for a potential clinical application to classify/predict the risk of graft rejection in transplant recipients. On the other hand, it may also facilitate in research applications the identification of putative analytes corresponding to discriminative peaks which, in turn, could accelerate the discovery of potential biomarkers that are sorely lacking in the context of both early type 1 diabetes pathogenesis and pancreatic islet transplantation.

## Conclusions

There is currently a critical need for reliable early biomarkers of type 1 diabetes pathogenesis and immune reactivity against allogeneic pancreatic islet transplants. Omics-type studies promise to contribute such biomarkers, but the identification of reliable biomarkers from single omics approaches has been challenging. Addressing this will likely require multi-omics approaches to identify rather comprehensive biomarker signatures that will be extracted from integrated multi-omics datasets generated in samples directly related to the tissue of interest. As demonstrated by Kantzelmeyer and colleagues, combined omics studies in such samples increased their ability to discriminate between kidney transplant recipients with and without cABMR [28]. It should be noted, however, that performing such studies in the context of diabetes and islet transplant has been more challenging due to the difficult access to samples that would be enriched with islet-related features, until now. In the present study, we demonstrated the proof-of-concept that combining the ACE-platform with the sensitive method of MEKC-LIFD to detect biochemicals/metabolites in microliter-size aqueous humor samples, we were able to track at least 13 distinctive biochemical features in association with the islet transplant outcome (i.e., rejection versus tolerance), and that could potentially serve as associated biomarkers. We also demonstrated the feasibility of machine training with only 22 samples to discriminate with better than 95% accuracy among animals that either rejected or tolerated their islet allografts and those that did not receive a transplant. We could also discriminate with 100% accuracy between the rejector and non-rejector recipients.

These findings warrant additional studies for building a larger electropherogram database to further improve the prediction accuracy among the three sample categories and to enhance and facilitate the identification of potential biomarker candidates that can then be further investigated and validated in the context of diabetes and islet transplantation [13, 14]. They also leave open the possibility of using the current paradigm to expand on the application of AI/ML to integrated multi-omics studies performed in aqueous humor samples that are enriched with islet features when islets are transplanted in the ACE [13, 15, 19]. Furthermore, the discovery of biomarker signatures using this experimental approach and their further validation in systemic and clinically relevant samples may ultimately improve the early detection of type 1 diabetes in at-risk subjects and the reliable classification of progressors from non-progressors in the clinical setting.

## Supporting information

S1 File (ZIP)
